# Prevalence and early outcome of bronchiectasis as an atypical presentation in COVID-19 patients

**DOI:** 10.1186/s43168-022-00164-4

**Published:** 2022-11-18

**Authors:** Aliae A. R. Mohamed Hussein, Ahmad M. Shaddad, Maiada K. Hashem, Maha Ahmed Okasha

**Affiliations:** 1 Chest Department, Faculty of Medicine, Assiut University, Assiut, Egypt; 2 Assiut Police Hospital, Assiut, Egypt

**Keywords:** COVID-19, Bronchiectasis, De novo, Prevalence, Outcome

## Abstract

**Introduction:**

Bronchiectasis was considered as an uncommon radiological feature of corona virus disease 2019 (COVID-19) infection. The clinical course and outcome of COVID-19 bronchiectasis overlap is still a point for research. The aim of this study was to evaluate the prevalence, course, and outcome of bronchiectasis as an atypical presentation of COVID-19 infection.

**Methods:**

A cross-sectional study has been conducted from July 2021 to February 2022 and included 425 COVID-19 swab-positive patients who were examined by high resolution computed tomography of the chest during acute phase (4 weeks) of the infection.

**Results:**

Fourteen (3.3%) patients newly developed bronchiectasis-de novo. Patients with de novo bronchiectasis had significantly higher cough score, frequency of colored sputum and mMRC score, respiratory distress (*p* < 0.001) and respiratory failure (*p* = 0.02) than patients with no bronchiectasis. They also had the higher frequency of ICU’s admission (*p* = 0.02), need to non-invasive (*p* = 0.01), and invasive mechanical ventilation (*p* = < 0.001), duration of mechanical ventilation, ICU’s stay and overall hospital stay (*p* < 0.001). As for the outcome, death rate was also statistically significantly higher among those with De novo bronchiectasis than those without bronchiectasis (*p* = 0.04).

**Conclusion:**

Bronchiectasis is an uncommon presentation among COVID-19 patients. However, bronchiectasis increases disease burden in COVID-19 patients. It may have a negative impact on the outcome.

**Trial registration:**

ClinicalTrials.gov. NCT04910113. Registered June 2, 2021.

## Introduction

Since February 2020 World Health Organization (WHO) named the novel variant coronavirus that was detected from the bronchoalveolar lavage of some patients suffering from severe lower respiratory tract infection in Wuhan City, China, in December 2019 [1] as severe acute respiratory syndrome coronavirus-2 (SARS-CoV-2). WHO called this infection Coronavirus Disease 2019 (COVID-19) which was declared on March 11th, 2020, as a global pandemic. On 18 April 2022, over 500 million confirmed cases and over 6.19 million deaths have been reported globally [2].

The typical chest CT appearance of COVID-19 pneumonia is bilateral peripheral ground-glass opacities (GGO), sometimes with areas of consolidation, with a prominent lower lung distribution [3, 4].

Bronchiectasis is a chronic lung condition, characterized clinically by dyspnea, productive cough, and recurrent respiratory infections, and radiologically by abnormal and permanent dilatation of the bronchi. The main radiological findings detected in chest CT of bronchiectasis are (1) internal diameter of a bronchus is wider than its adjacent pulmonary artery; (2) loss of bronchial tapering; and (3) extension of visualized dilated bronchi into the outer cortical lung areas [4].

One of the reported radiological findings in patients with COVID-19 infection was bronchiectasis. However, published studies lacked reporting the onset and evolution of bronchiectasis during the short term follow-up as well as long-term complications among those patients [5].

Traction bronchiectasis is a common variant of bronchiectasis which can be described as bronchial dilatation secondary to mechanical traction from adjacent pulmonary fibrosis. Pathogenesis was assumed to occur during severe COVID-19. In addition, lung injury secondary to mechanical ventilation due to sever infection could be added to this theory [6].

This study aims to evaluate the prevalence and pattern of bronchiectasis as an atypical presentation of COVID-19 infection and to assess the course and outcome of COVID-19 infection presented with bronchiectasis during the acute phase of the disease.

## Patients and methods

A cross-sectional study has been conducted at Assiut University and Assiut Police Hospitals from July 2021 to February 2022. Patients who were enrolled in this study were either from patients admitted to the hospitals or those who went to the outpatient’s clinics. Diagnosis of COVID-19 infection among the participants was established according to the WHO and Egyptian Ministry of Health and Population (MOHP) guidelines [7, 8]. The cornerstone for diagnosis were RT-PCR and MSCT chest. Case detection was done using RT-PCR for the viral RNA by TaqMan™ 2019-nCoV Control Kit v1 (Cat. No. A47532) supplied by QIAGEN, Germany on the Applied Biosystem 7500 Fast RT PCR System, USA. All patients admitted during the study period in study hospitals with confirmed COVID-19 infection and met the inclusion criteria were included in this study.

The inclusion criteria were adult patients aged 18 years or older, of both genders who were diagnosed as COVID-19-positive by RT-PCR in Assiut University and Assiut Police hospitals during the study period who had chest CT scan examination during acute stage of the diseases. Children under 18 years old, patients who were not examined by CT scan of the chest and those who refused to participate in the study were excluded.

### Data collection

All patients included in this study were subjected to detailed history and physical examination, relevant laboratory investigations such as complete blood picture, inflammatory markers, and arterial blood gases, as well as high-resolution CT chest.

Regarding the symptoms evaluation, dyspnea was assessed by modified medical research council (mMRC) score while cough was assessed by Cough Symptom Score (CSS) which is a simple a two-part questionnaire referring to daytime and night-time symptoms. Based on the frequency, intensity and influence of cough on daily activities and sleep, cough symptoms are scored from 0 to 5, with 0 indicating no cough and 5 indicating the most severe cough [9].

The computed tomography was performed using GE Optima 64 slices made in the USA. Patients were examined in supine position. The scanning extended superiorly from the thoracic inlet and ended by the diaphragm. No contrast media injection was used in either patient. All CT examinations were reviewed and reported by the attending radiologists who were blinded to the study. CT sections of all patients were examined for the presence and distribution of any of the following: ground-glass opacities (GGO), consolidation, multifocality, distribution (peripheral or diffuse), septal thickening, crazy paving, pulmonary nodules, pleural effusion, and mediastinal lymph nodes for the diagnosis of COVID-19 infection and assessment of its severity [3, 10]. They also were assessed for the presence of any increase in bronchial to adjacent pulmonary artery diameter ratio, loss of the bronchial tapering, and visualization of bronchi in the outer 1–2 cm of the lung fields for the diagnosis of bronchiectasis with evaluation of its anatomical distribution [11].

Based on the clinical and radiological evidence of bronchiectasis diagnosis patients were divided into three groups:

- Group I included COVID-19 patients without bronchiectasis.
- Group II included COVID-19 patients who newly developed bronchiectasis-de novo.
- Group III included COVID-19 patients with preexisting chronic bronchiectasis. Group III were then excluded from further statistical analysis in this study.

### Ethical considerations

The study was approved by Assiut University, Faculty of Medicine ethical committee under (IRB no. 17101456). The singed informed consent form was a permanent part of the participants study records, and it was maintained in the same manner as other records. Patients’ identity and personal information were concealed, and each patient assigned for a code to insure privacy and confidentiality of the data. It was performed according to the provisions of the Declaration of Helsinki. The study was also registered in clinicaltrials.gov under number (NCT04910113).

### Statistical analysis

Data was collected and analyzed by using SPSS (Statistical Package for the Social Science, version 20, IBM, and Armonk, New York). The Shapiro test was used to determine compliance of the data to normal distribution. All quantitative data in the current study had abnormal distribution and compared with Mann-Whitney test. Nominal data were given as number (*n*) and percentage (%). *Chi*^*2*^ test was implemented on such data. *P* value was considered significant if < 0.05.

## Results

A total of 425 patients with confirmed COVID-19 infection were enrolled in the current study. Based on development of bronchiectasis, those patients were subdivided into the following groups:

- Group I: COVID-19 patients without bronchiectasis, 400 (94.1%) patients,
- Group II: COVID-19 patients who newly developed bronchiectasis- De novo, 14 (3.3%) patients, and
- Group III: COVID-19 patients with preexisting chronic bronchiectasis, 11 (2.6%) patients. Group III were then excluded from further statistical analysis in this study.

**Fig. 1:**
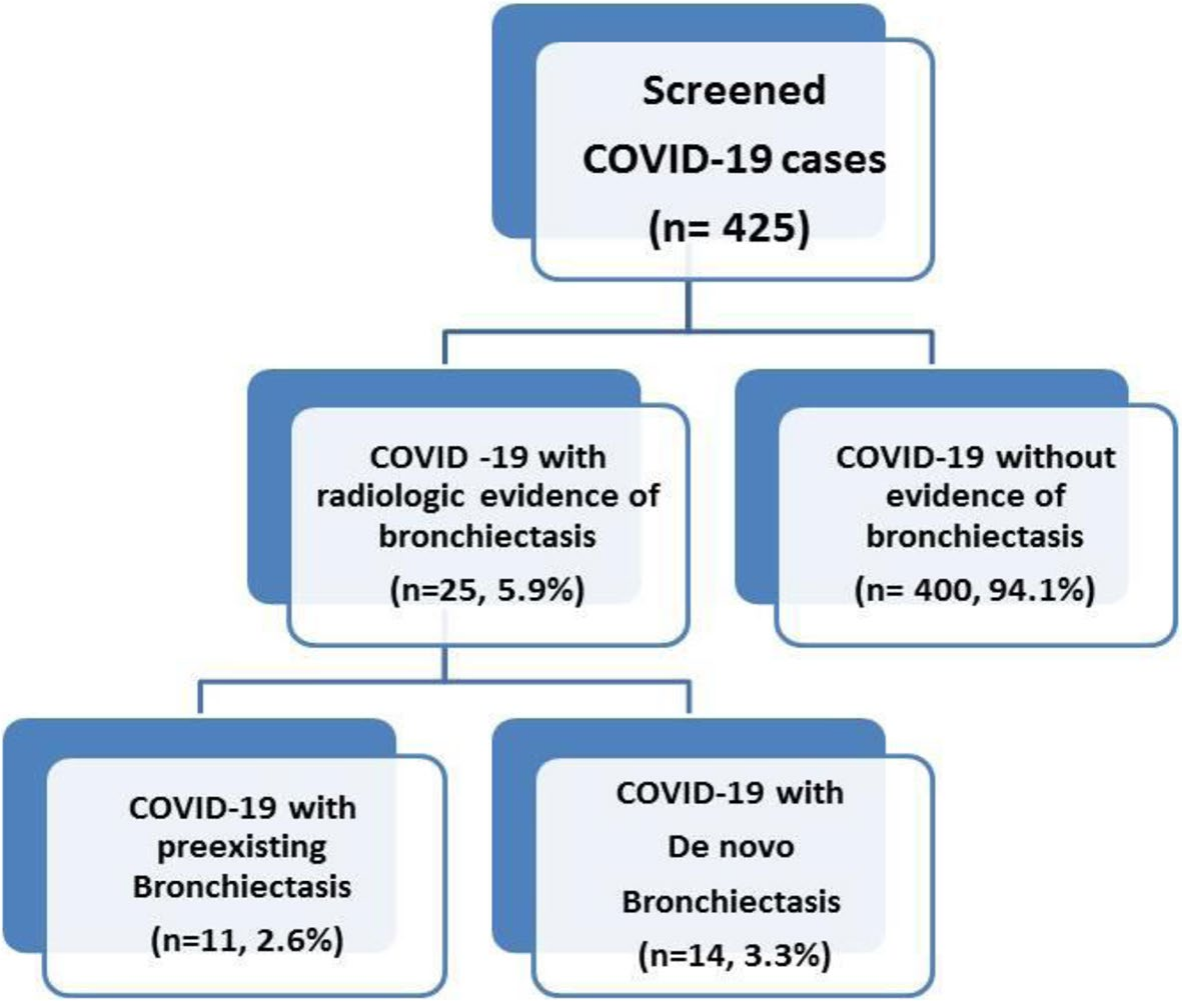
Flowchart of screened COVID-19 cases included in the study (*n* = 425)

The flowchart of recruited cases is shown in (Fig. 1).

The mean age of the patients in the two groups were 52.62 ± 17.80 and 57.64 ± 10.96 years respectively and more than half of them were males with no statistically significant difference. Patients with de novo bronchiectasis had significantly higher frequency of COPD in comparison to those without bronchiectasis (42.9% vs. 6.5%; *p* < 0.001) (Table 1).

**Table 1:** Demographic characteristics of COVID-19 patients without and with de novo bronchiectasis included in the study

	COVID-19 without bronchiectasis (*n* = 400)	COVID-19 with de novo bronchiectasis (*n* = 14)	***p***
Age (years)	52.62 ± 17.80	57.64 ± 10.96	0.30
Sex			0.45
Male	257 (64.3%)	10 (71.4%)	
Female	143 (35.8%)	4 (28.6%)	
Active smoking	323 (80.8%)	5 (35.7%)	**< 0.001**
Comorbidities			
COPD	26 (6.5%)	6 (42.9%)	**< 0.001**
Bronchial asthma	5 (1.3%)	0	0.84
Diabetes mellitus	76 (19%)	2 (14.3%)	0.49
Hypertension	106 (26.5%)	1 (7.1%)	0.08
Hepatic disease	2 (0.5%)	0	0.93
Renal disease	14 (3.5%)	0	0.61
Cardiac diseases	29 (7.2%)	1 (7.1%)	0.37
Other	28 (7%)	3 (21.4%)	0.07

Data expressed as frequency (percentage), mean (SD). *P* value was significant if < 0.05 *COPD* chronic obstructive pulmonary disease, *COVID-19* coronavirus disease 2019

Age was compared by Mann-Whitney test and all nominal data was compared by chi^2^ test

Patients with de novo bronchiectasis had significantly higher cough score (4.93 ± 0.27 vs. 3.05 ± 1.88; *p* < 0.001), higher frequency of colored sputum (72.7% vs. 18.8%; *p* < 0.001) and higher mMRC dyspnea score in comparison to those without bronchiectasis. Moreover, patients with de novo bronchiectasis had significantly higher frequency of respiratory distress (50% vs. 14%; *p* < 0.001) and respiratory failure (78.6% vs. 49.5%; *p* = 0.02) than those without bronchiectasis, respectively (Table 2).

**Table 2:** Clinical characteristics of COVID-19 patients without and with de novo bronchiectasis included in the study

	COVID-19 without bronchiectasis (*n* = 400)	COVID-19 with de novo bronchiectasis (*n* = 14)	*P*
Cough	338 (84.5%)	14 (100%)	0.33
Cough score	3.05 ± 1.88	4.93 ± 0.27	**< 0.001**
Sputum			**< 0.001**
None	309 (77.3%)	2 (14.3%)	
Whitish	16 (4%)	1 (1.7%)	
Colored	75 (18.8%)	11 (72.7%)	
Blood tinged	0	0	
Dyspnea	294 (73.5%)	13 (92.9%)	0.08
mMRC score			**0.04**
0	137 (34.3%)	1 (7.1%)	
1	5 (1.3%)	0	
2	53 (13.3%)	0	
3	4 (1%)	0	
4	201 (50.2%)	13 (92.9%)	
Fatigue	393 (98.3%)	14 (100%)	0.78
Sore throat	123 (30.8%)	3 (21.4%)	0.33
Cyanosis	199 (49.8%)	10 (71.4%)	0.09
Respiratory distress	56 (14%)	7 (50%)	**< 0.001**
Respiratory failure	198 (49.5%)	11 (78.6%)	**0.02**

Data expressed as frequency (percentage), mean (SD). *P* value was significant if < 0.05

*mMRC* modified medical research council, *COVID-19* coronavirus disease 2019

Cough score was compared by Mann-Whitney test and all nominal data was compared by chi^2^ test

Serum ferritin was significantly higher among those with De novo bronchiectasis in comparison to those without bronchiectasis (1392.97 ± 969.32 vs 489.74 ± 222.34; *p* < 0.001). In contrast, oxygen saturation at admission was significantly lower among those with de novo bronchiectasis in comparison to those without bronchiectasis (62.29 ± 15.67 vs. 84.38 ± 14.10; *p* < 0.001) (Table 3).

**Table 3:** Laboratory findings of COVID-19 patients without and with de novo bronchiectasis included in the study

	COVID-19 without bronchiectasis (*n* = 400)	COVID-19 with de novo bronchiectasis (*n* = 14)	*P*
Leucocytes (10^3^/μl)	9.47 ± 4.01	11.12 ± 5.32	0.13
Lymphocytes (10^3^/μl)	1.18 ± 0.68	0.86 ± 0.47	0.09
CRP (mg/dl)	51.64 ± 34.45	72.38 ± 52.69	0.21
D-dimer (ng)	1.45 ± 1.22	1.93 ± 0.93	0.61
Serum ferritin	489.74 ± 222.34	1392.97 ± 969.32	**< 0.001**
SO_2_ at admission (%)	84.38 ± 14.10	62.29 ± 15.67	**< 0.001**
SO_2_ at discharge (%)	88.78 ± 19.90	93.88 ± 1.47	0.53

Data expressed as frequency (percentage), mean (SD). *P* value was significant if < 0.05 *SO*_*2*_ oxygen saturation, *COVID-19* coronavirus disease 2019, *CORAD* COVID-19 Reporting and Data System, *CRP* C-reactive protein

All data was compared by Mann-Whitney test and CORAD class was compared by chi^2^ test

Regarding the course of COVID-19 infection among both groups, the frequency of ICU’s admission (64.3% vs. 25.3%; *p* = 0.02), need to non-invasive (50% vs. 20.3%; *p* = 0.01), and invasive mechanical ventilation (50% vs. 12.8%; *p* = < 0.001), duration of mechanical ventilation, ICU’s stay, and overall hospital stay were all statistically significantly higher among those with de novo  bronchiectasis than those without bronchiectasis (*p* < 0.001). As regards the outcome, death rate was also statistically significantly higher among those with de novo bronchiectasis than those without bronchiectasis (14.8% vs. 35.7%; *p* = 0.04) (Table 4).

**Table 4:** Clinical course and outcome of COVID-19 patients without and with de novo bronchiectasis included in the study

	COVID-19 without bronchiectasis (*n* = 400)	COVID-19 with de novo bronchiectasis (*n* = 14)	*P*
Management site			**< 0.001**
At home	195 (48.8%)	3 (21.4%)	
At the ward	104 (26%)	2 (14.3%)	
ICU	101 (25.3%)	9 (64.3%)	
Hospital stay (day)	4.33 ± 2.15	25.14 ± 10.98	**< 0.001**
ICU’s stay (day)	1.79 ± 1.32	11.07 ± 10.45	**< 0.001**
Ventilatory support			
NRM	73 (18.3%)	2 (14.3%)	0.51
Vapotherm	25 (6.3%)	1 (7.1%)	0.61
Non-invasive ventilation	81 (20.3%)	7 (50%)	**0.01**
Mechanical ventilation	51 (12.8%)	7 (50%)	**< 0.001**
Duration of MV (day)	3.57 ± 1.23	14.13 ± 7.57	**< 0.001**
Outcome			
Alive	341 (85.3%)	9 (64.3%)	**0.04**
Death	59 (14.8%)	5 (35.7%)	

Data expressed as frequency (percentage), mean (SD). *P* value was significant if < 0.05 *ICU* intensive care unit, *NRM* non-rebreathing mask, *MV* mechanical ventilation, COVID-19 coronavirus disease 2019

Continuous data was compared Mann-Whitney test and all nominal data was compared by chi^2^ test

## Discussion

This cross-sectional study included 425 patients with COVID-19 infection confirmed by PCR in duration between July 2021 and February 2022. Patients were subdivided into three groups according to the presence or absence of bronchiectasis, COVID19 patients without bronchiectasis (group I) (94.1%), COVID19 patients developing bronchiectasis (group II) (3.3%) and bronchiectasis patients with COVID19 (group III) (2.6%).

A Chinese nationwide retrospective cohort studied the prevalence of chronic respiratory diseases among 39,420 laboratory-confirmed COVID-19 patients and its outcome from the electronic medical records, found that bronchiectasis was present in 27.9%. The association between COPD and bronchiectasis was found in 50.7% while association asthma and bronchiectasis was found in 15.9% of those patients, respectively [12]. On the other hand, Ambrosetti and colleagues detected two cases of lower lung lobe bronchiectasis developed in a few days and confirmed by CT in two patients with severe respiratory symptoms due to COVID-19 infection without significant comorbidities [13].

Another retrospective study analyzed the radiological finding of 81 patients with COVID-19 detected bronchiectasis in nine (11%) out of all included patients. The incidence of bronchiectasis development was increased with disease progression according to that study [14].

Furthermore, out of 101 COVID-19 positive cases examined in Zhao and colleagues’ retrospective study 53 patients (52.5%) had traction bronchiectasis, similar to that seen post SARS [15].

By comparing 8070 patients with a confirmed diagnosis of COVID-19 after January 2020 with the matched 121,050 age-, sex-, and residence non COVID participants registered in the Korean National Health Insurance Service (NHIS), the rate of bronchiectasis was 1.6% in the COVID-19 group and 1.4% in the matched group (*p* = 0.003) with a 1.22-fold increased odds ratio of the prevalence of bronchiectasis in the COVID-19 group relative to the matched group [16].

Another 6 months longitudinal study among 114 survivors after severe pneumonic type COVID-19 infection revealed a radiological evidence of bronchiectasis development in up to 24% of the patients at the follow up 6 months period compared with only 7% in the acute stage of the disease. These findings suggest that bronchiectasis (at least from a radiological point of view) can appear in up to a quarter of patients who suffered from severe COVID-19 pneumonia [17]. Moreover, Hanley et al. observed traction bronchiectasis in 53% of cases in a postmortem CT study out of 101 bodies. The radiological involvement had bilateral lower lobes predominance [18].

The pathogenesis of bronchiectasis 2ry to COVID-19 infection is relatively unknown especially in acute stage of the infection. However, with disease progression, in many cases dilatation may be due to traction in the context of pulmonary fibrosis. From this prospective, Han et al. observed that most of patients presented with bronchiectasis also had pulmonary fibrotic changes secondary to the COVID-19 pneumonia [17]. In this respect, the nature of the inflammatory component of this bronchiectasis, its potential to lead to recurrent bronchial infection and inflammation by potentially pathogenic microorganisms and, therefore, its potential to worsen prognosis, are unknown [19].

In regard to the demographic data of patients included in the current study, patients with de novo bronchiectasis had significantly higher frequency of COPD in comparison to those without bronchiectasis. All other comparisons including age, sex, and other comorbidities such as hypertension, diabetes mellitus, renal, and cardiac diseases were statistically insignificant. In a previous large series, COVID-19 patients with bronchiectasis were significantly older with more frequent pulmonary comorbidities, such as asthma (62.1% versus 30.4%) and COPD (57.6% versus 17.8%), than those without bronchiectasis. Moreover, extra-pulmonary comorbidities were more frequent in patients with bronchiectasis, such as hypertension (52.3% versus 27.0%), diabetes mellitus (61.4% versus 31.0%), and heart failure (27.3% versus 10.1%), than those without bronchiectasis (*p* < 0.001 for all) [16]. Han et al. observed that age greater than 50 years was a prognostic factors for subsequent fibrotic sequalae [17].

Patients with de novo bronchiectasis had significantly higher cough score and frequency of colored sputum production in comparison to those without bronchiectasis in the current study which could be explained by the pathological nature of bronchiectasis as a suppurative disease characterized by chronic cough and sputum production due to impaired mucocilliary clearance [20].

Also, patients with de novo bronchiectasis had significantly higher frequency of respiratory failure and lower oxygen saturation at time of admission in comparison to those without bronchiectasis in the current study. Hypoxemia may be developed in those groups due to fibrotic changes which lead to traction bronchiectasis or due to chronic obstructive lung disease which affect lung function [21, 22].

There was also significant elevated serum ferritin level among those with de novo bronchiectasis in comparison to those without bronchiectasis. This result can be explained by ferritin contribution to increased production of free radicals and hence oxidative tissue damage. Ferritin level is concomitant with both acute and chronic inflammatory processes. Thus, elevated ferritin that reflects persistent inflammatory reactions that can cause structural damage of the pulmonary parenchyma 2ry to COVID-19 infection [23].

The frequency of ICU’s admission, need to non-invasive and invasive mechanical ventilation, duration of mechanical ventilation, ICU’s stay, and overall hospital stay were all statistically significantly higher among those with De novo bronchiectasis than those without bronchiectasis in the current study. Two cases of COVID associated bronchiectasis in Ambrosetti et al. study presented by respiratory failure, elevation of CRP and D-dimer, and need ICU admission for non-rebreathing mask or non-invasive ventilation [5]. Han et al. also observed that the median hospital stay was longer for COVID-19 participants who developed bronchiectasis than those who did not (27 days [IQR, 26] vs 15 days [IQR, 8], *P* 0.001) [17]. Moreover, Suliman and colleagues reported a patient who developed progressive bronchiectasis 4 weeks after the onset of severe COVID-19 pneumonia. This patient needed ICU admission for invasive ventilation with long hospital stay (39 days) and long duration of PCR conversion as well [24].

Choi et al. observed that COVID-19 patients with bronchiectasis suffered from more severe infection than those without bronchiectasis. In that study, COVID-19 patients with bronchiectasis had worse course and outcome regarding use of supplemental oxygen, ICU admission rates, need for ECMO, and higher mortality than those without bronchiectasis. Impaired mucociliary clearance and chronic inflammation in the airway of patients with bronchiectasis is likely to increase their susceptibility to and severity of COVID-19 [16].

Regarding the outcome, the current study revealed that death rate was significantly higher among those with de novo bronchiectasis than those without bronchiectasis.

To our knowledge, no previous studies had discussed the risk factors for bronchiectasis development in COVID-19 infection but advanced age, smoking, sever COVID-19 infection, high levels of inflammatory biomarkers and prolonged mechanical ventilation are reported risk factors for development of post-COVID-19 pulmonary fibrosis [25, 26].

The current study faced some limitations. First, the small number of recruited patients in a single city during a limited time frame resulted in a small number of patients who developed bronchiectasis. Thus, the accuracy of comparative statistics could not be fully guaranteed. Second, we evaluated the development of bronchiectasis in the acute stage of COVID-19 infection (4 weeks), however, the development of traction bronchiectasis in post-COVID fibrosis was not assessed. Third, the impact of different and widely variable antimicrobial, anti-inflammatory, and immunosuppressive drugs used in the treatment of COVID-19 infection on development and progression bronchiectasis was not evaluated.

In conclusion, bronchiectasis is an uncommon presentation among COVID-19 patients. However, bronchiectasis increases disease burden in COVID-19 patients. It may have a negative impact on the outcome. Awareness must be raised among physicians and radiologists about bronchiectasis as an atypical presentation for COVID-19 infection. Moreover, bronchiectasis could be a possible common sequel for COVID infection. Thus, screening of bronchiectasis in survivors of COVID-19 infection especially those with chronic pulmonary symptoms or recurrent respiratory tract infection should be recommended.

## Data Availability

The datasets analyzed during the current study are available upon request.

## Abbreviations

COVID-19: Corona Virus Disease 2019
ECMO: Extra-corporeal membrane oxygenation
GGO: Ground glass opacity
HRCT: High-resolution computed tomography
mMRC: Modified Medical Research Council
SARS-CoV-2: Severe acute respiratory syndrome coronavirus-2
